# Attention deficit/hyperactivity disorder (ADHD) dimensions mediate the relationship between adverse childhood experiences and adult aggression depending on cognitive reappraisal

**DOI:** 10.1038/s41598-025-87861-4

**Published:** 2025-01-30

**Authors:** Steffen Barra, Paulina Klaudia Machalica, Petra Retz-Junginger, Johannes Merscher, Anselm Crombach, Wolfgang Retz

**Affiliations:** 1 Institute for Forensic Psychology and Psychiatry, Saarland University, Homburg, Germany; 2 Department of Psychology, Saarland University, Saarbruecken, Germany; 3 Department of Psychiatry and Psychotherapy, University Medical Center of the Johannes Gutenberg-University Mainz, Mainz, Germany

**Keywords:** Social behaviour, Human behaviour

## Abstract

Associations between adverse childhood experiences (ACEs) and aggressive behavior have often been demonstrated, but the mechanisms underneath these relations are yet unclear. As high levels of ACEs and aggression have been found among individuals with attention deficit/hyperactivity disorder (ADHD), ADHD dimensions might explain this association. Moreover, maladaptive emotion regulation is common in ADHD and was associated with aggressive behavior. The present study investigated the dynamics among these constructs in a mixed sample of 287 adults. We found partially mediating effects of current ADHD on the associations of ACEs with adult aggression, especially regarding the hyperactive/impulsive dimension. Cognitive reappraisal moderated the indirect effect between hyperactivity/impulsivity and aggression, especially for females. Hence, the unfavourable dynamics found in the present study might explain the increased risk for aggressive behavior in individuals affected by both, ACEs and ADHD. Respective gender-sensitive prevention and treatment for aggressive behavior should include adequate pharmacological and psychological approaches that address ADHD core symptoms, whilst also aiming to improve emotion regulation techniques.

## Introduction

Adverse childhood experiences (ACEs) reflect a variety of dysfunctional influences occurring during childhood and adolescence, including physical, emotional, or sexual abuse, neglect, but also further household dysfunction, and difficult peer experiences. ACEs have been consistently associated with the potential to negatively affect development until and throughout adulthood^1^. Depending on the type of ACE assessed, recent international studies point to prevalence rates of 12% (sexual abuse) to 36% (emotional abuse) among minors and 4% (sexual abuse/physical neglect) to 13% (emotional neglect) among adults with a tendency of females reporting higher ACE burden than males^2,3^. Moreover, research has found that a considerable number of people not only had to face one, but multiple ACEs^1^. Meta-analytic evidence highlights the disadvantageous long-term effects of (cumulative) ACEs on health and behavior, especially regarding risk of mental health problems and self-directed as well as interpersonal aggression^4^.

Among psychiatric conditions, both ACEs and aggressive tendencies are highly prevalent in externalizing disorders such as conduct disorder and attention-deficit/hyperactivity disorder (ADHD)^5–7^. ADHD is one of the most common psychiatric disorders in childhood and adolescence with prevalence rates between 5 and 7%^8,9^. About 50 to 80% show clinically relevant symptoms up to adulthood, whereas in about 15% of all cases, full ADHD symptomatology remains^10,11^. Although ADHD is more often diagnosed in males than in females, concern has been expressed that ADHD is understudied and frequently overlooked in females, e.g., due to somewhat different appearances of respective symptoms and gender bias^12,13^.

Core symptoms of ADHD include inattention, impulsivity, and hyperactive behavior. Assessment of adult ADHD is often complicated by the fact that some of the prominent symptoms of childhood ADHD, such as hyperactivity, may be rather hidden, e.g., in terms of an inner tension^11,14^. Moreover, ADHD is often accompanied by several comorbid mental health problems and functional impairments that may show some overlap with actual ADHD symptoms, further impeding clear and distinct differentiation^15–17^. DSM-5 and ICD-11 allow specifying ADHD according to the most prevalent symptom clusters as mainly inattentive, mainly hyperactive/impulsive, or combined. ADHD symptomatology is considered fluctuating over the lifespan; thus, changes in ADHD presentation may occur from childhood over adolescence into adulthood^18^. A dimensional perspective on ADHD symptomatology appears fruitful in order to consider the wide heterogeneity and different symptom severity but also possible distinct associations with further impairments among ADHD patients^19^.

Unfavourable dynamics among ACEs, ADHD, and aggression appear probable, as both ACEs and ADHD display risk factors for aggressive behavior, especially in combination^3,20^. Risk factors for aggressive behaviour such as respective cognitive schemes, negative attitudes, and deficient empathy are commonly stated among individuals burdened by ACEs^21^. As ADHD is defined as a neurodevelopmental disorder in DSM-5 with an onset in childhood, it can be assumed that childhood adversity such as ACEs may be related to the development of ADHD symptoms. Craig et al.^6^ have summarized the current knowledge on the dynamics between ACEs and ADHD. According to their literature review, the experience of ACEs has been linked to increased risk of clinically relevant ADHD and comorbid externalizing symptomatology. Although this relationship appeared to be robust in cross-sectional studies, longitudinal findings were less consistent. For children and adolescents, however, there was also the possibility of bidirectional associations, as ADHD symptoms may foster maladaptive parental reactions resulting in ACEs.

Yet, when focusing on adult ADHD dimensions, bidirectional associations with ACEs appear less plausible, because ACEs by definition occur prior to adulthood. Indeed, ACEs and associated stress may further increase attention and hyperactivity/impulsivity problems in (adult) people already dealing with ADHD symptomatology^22,23^, thus leading to greater risk of aggressive behavior^20,24^. Previous research has indicated that ACEs promote alterations in both structure and functioning of systems associated with stress management and executive function, such as the hippocampus, amygdala, and prefrontal cortex, which are often found impaired in ADHD patients, too^25^.

Aggressive behavior as a consequence of the inattention dimension of ADHD may result from failures to adequately perceive or process social cues, thus increasing the risk of aggression promoting cognitive phenomena such as hostile attribution bias^26^. Problems regarding hyperactivity/impulsivity may particularly favor the occurrence of reactive forms of aggression as a thoughtless and unpremeditated response to perceived threat or frustration based on dysfunctional neurobiological, cognitive, and emotional threat sensitivity or stress processing^27,28^. Although patients with mainly hyperactive/impulsive symptoms had shown greater risk of aggression than those with mainly attention deficits^20,29^, both dimensions may also operate in combination, e.g., when hostile attribution or missed information based on inattention foster feelings of threat or frustration that provoke aggressive behavioral responses facilitated by hyperactivity/impulsivity^30^. Along with these assumptions, not only ACE, but also ADHD prevalence is, regardless of gender, higher in both juvenile and adult criminal (violent) offender samples than in the community and has been associated with early, severe, and chronic offending histories^5,31,32^.

A further common impairment associated with ADHD is emotional dysregulation, which is often characterized by the experience of unpredictable changes in emotions and temper, giving rise to an ample debate about the need to include emotional dysregulation as a specific domain of ADHD psychopathology^33^. In their recent review, Bodalski et al.^34^ pointed out that emotional dysregulation in ADHD may, among others, depend on gender (with females showing higher rates of emotional dysregulation than males) and core symptom severity but may also be due to the reduced ability to use adequate strategies to cope with certain emotional states. Maladaptive emotion regulation or coping describes deficiencies in the ability to maintain or change current positive or negative emotional states. Whereas adaptive emotion regulation strategies such as acceptance, problem solving, and reappraisal seem to have favourable effects on psychological wellbeing, maladaptive coping in terms of avoidance, rumination, and suppression was associated with higher general and ADHD associated psychopathology^34,35^. Moreover, dysfunctional emotional coping has been linked to aggressive behavior^36^.

Gross^37^ differentiated emotion regulation techniques by the timing of occurrence in preparation and response strategies: Preparation strategies are applied before a certain emotional state has been established and/or a specific emotional reaction has been shown. Response strategies aim at modifying emotional states or reactions that have already occurred. One of the most prominent preparation strategies is cognitive reappraisal, whereas suppression is most often evaluated as response strategy^38^. Reappraisal serves to assign a subjective meaning to an emotional event in advance to modify the interpretation of a potentially emotional consequence, eventually impeding the risk of negative affect; suppression, however, is aimed at disguising a certain behavior or expression after an emotional event has occurred in such a way that the actual emotional state remains unknown to others^39^. In their literature review, John and Gross^40^ summarized that reappraisal led to decreased feelings and expressions of negative emotions, whereas suppression was associated with reduced expression but unchanged feelings of negative emotional states. Furthermore, reappraisal had favourable influences on cognitive and social functioning while positively affecting overall well-being on the long run. In contrast, suppression allowed to maintain a short-term positive emotional state but reduced overall well-being over time. Roberton et al.^36^ stressed that suppression may promote aggressive tendencies, e.g., anger, which may lead to increased readiness to act out aggressively. A current systematic review concluded that despite mixed results, cognitive reappraisal was commonly associated with lower, but suppression with higher risk of aggression^41^. Regarding gender differences, prior studies not only stressed that women and men differed in the frequency of use of reappraisal and suppression, but that both strategies showed gender-specific associations with aggressive tendencies^42^.

Although distinct links among ACEs, ADHD, aggression, and emotion regulation strategies have been examined^4,20,36^, to the best of our knowledge, studies are lacking which consider all those variables simultaneously. Further examining the dynamics among these constructs may broaden our understanding of the emergence of aggressive behavior and may inspire the development of prevention and intervention approaches to address people at risk. Testing mediation models and including emotion regulation strategies as moderators may shed light on the role of distinct ADHD dimensions and emotional coping mechanisms in the interplay between ACEs and adult aggression and allow clinical implications that consider both biographical (trauma-oriented) and ADHD-focused treatment options to reduce the risk of adult aggression, potentially interrupting a cycle of violence.

In addition, facing the already identified gender-related specifics regarding these constructs, respective research should account for differences between genders. Hence, the present study aimed to contribute to this lack of research by examining the associations among ACEs, ADHD, aggression, and emotion regulation strategies in a mixed adult sample. Based on previous research, we assumed that the severity of ACEs experienced in childhood and adolescence would be positively associated with aggressive behavior in adulthood. Second, we proposed that this effect would be partially mediated by adult ADHD symptomatology. We expected a greater effect regarding the hyperactive/impulsive compared to the inattentive dimension. Furthermore, we assumed that the relations among ACEs, ADHD, and aggression would be moderated by emotion regulation strategies: reappraisal should reduce, suppression should increase these associations. Differences between males and females were examined in an exploratory manner.

## Methods

### Procedures

The current study was conducted within the framework of a larger examination of clinically and forensically relevant risk factors in a mixed sample of individuals who had been examined at the Institute for Forensic Psychology and Psychiatry, Saarland University, Homburg, for either forensic evaluation/therapy or ADHD assessment. As part of the general assessment procedures, a standardized self-report questionnaire set was filled out including the measurements considered for this study (see below). Additionally, students and interns affiliated to the abovementioned institute recruited a non-forensic/non-clinical sample by personal addressing of acquaintances. Questionnaires were offered in either paper–pencil or digital format (www.soscisurvey.de). By involving forensic, clinical, and (healthy) control participants, we aimed at increasing the heterogeneity and, thus, the variance in our constructs of interests. Individuals were free to decide whether their anonymized data would be used for research purposes. Informed consent was obtained. Study participation was voluntary and unpaid. Study procedures were performed in accordance with the ethical standards of the Declaration of Helsinki and approved by the ethics committee of the Medical Chamber of Saarland, Germany (protocol code: 58/22).

### Participants

A total of 352 individuals were assessed between November 11th 2021 and December 5th 2023. However, 7 refused to have their data analysed for research purposes and 4 did not answer this question. Of the remaining 341 participants, a total of 287 had filled out the below mentioned questionnaires of interest for the present study. In this final sample, 130 individuals identified as male (45.5%), 154 as female (53.7%), and 3 as diverse (1.0%). Due to the limited number of diverse participants, we only conducted gender specific analyses with the data of male and female participants, but included those of diverse participants when gender was controlled. Age at assessment ranged between 18.9 and 72.9 years (M = 34.9 years, SD = 12.7 years). Sixty-six (23.0%) participants had been seen at the institute for forensic evaluation/therapy and 89 (31.0%) for ADHD assessment. Moreover, 132 (46.0%) control participants took part in the study.

### Measurements

#### Adverse childhood experiences

ACEs were assessed by the German 75-item version of the Maltreatment and Abuse Chronology of Exposure (MACE) scale^43,44^. Using a dichotomous yes–no response format, participants were asked whether they had been exposed to events assignable to a total of 10 ACE categories up to the age of 18 years: verbal parental abuse, non-verbal parental emotional abuse, parental physical abuse, emotional neglect, physical neglect, witnessing violence towards/between parents, witnessing violence towards siblings, emotional abuse by peers, physical abuse by peers, and sexual abuse. After calculating subscale sum scores for each ACE category, interpolation led to comparable subscale scores between 0 and 10^43^. For the present study, a total MACE score was used by adding up all subscale scores, possibly ranging between 0 and 100. Higher scores indicated more severe ACE burden. Previous research has pointed to good psychometric properties of both the original and German MACE^43,44^.

#### Aggression

Recent aggression was measured by the aggressive behavior subscale of the German Adult Self-Report (ASR 18/59^45^). The ASR 18/59 contains a total of 126 items addressing several internalizing and externalizing problems during the past 6 months, of which 15 belong to the aggressive behavior subscale. Items are rated on a 3-point Likert-scale (0 = not true, 1 = somewhat or sometime true, 2 = very true or often true). Psychometric properties of the ASR 18/59 were proven in previous studies^46^. In the present sample, internal consistency for the aggressive behavior subscale was high with Cronbach’s α = 0.872.

#### ADHD

Current ADHD symptomatology was assessed by the German self-report questionnaire for adult ADHD (ADHS-SB^47^). The ADHS-SB contains a total 22 items to be rated on a four-point Likert-scale (0 = not applicable, 1 = slightly/occasionally, 2 = moderate/often, 3 = severe/almost always). Inattention is captured by the first 9 items, whereas the following 9 items relate to hyperactivity/impulsivity. The other 4 questions address functional impairments and childhood symptomatology. The ADHS-SB allows the deduction of clinically relevant ADHD according to DSM-5 presentations (mainly inattentive, mainly hyperactive/impulsive, combined) but also based on a total ADHD score with a proposed cut-off of 15 points. However, for the present study, we focused on a dimensional perspective on both inattention and hyperactivity/impulsivity using sum scores of the items 1–9 and 10–18, respectively. The psychometric properties of the ADHS-SB had been evaluated by the authors^48^. In the present sample, internal consistency was excellent with Cronbach’s α = 0.927 for the inattentive and α = 0.910 for the hyperactive/impulsive dimension.

#### Emotion regulation strategies

Emotion regulation strategies were assessed by the German version of the Emotion Regulation Questionnaire (ERQ^39,49^). The ERQ contains 6 items addressing cognitive reappraisal and 4 items relating to suppression. Items are rated on a 7-point Likert-scale (1 = strongly disagree to 7 = strongly agree). Mean values for each regulation strategy are built. Psychometric properties had been proven by the authors^39,49^. In the present study, internal consistencies were high for both reappraisal (Cronbach’s α = 0.872) and suppression (Cronbach’s α = 0.800).

#### Covariates

At the very beginning of the questionnaire set, individuals were asked to report their gender identification (male, female, or diverse) and their date of birth. The latter was used to calculate their age at the time of assessment.

### Statistical analyses

All statistical analyses were conducted in IBM SPSS Statistics (Version 28). The general level of significance was set at p < 0.05. Tests were conducted two-sided. Internal consistency was interpreted as questionable with Cronbach’s α ≥ 0.60, acceptable with α ≥ 0.70, good with α ≥ 0.80, and excellent with α ≥ 0.90^50^. We used MANOVAs for descriptive group comparisons between male and female participants; the effect size partial eta^2^ (η_p_^2^) indicated a small effect with η_p_^2^ ≤ 0.01, a moderate effect with η_p_^2^ = 0.06, and a large effect with η_p_^2^ = 0.14^51^. Furthermore, we examined (partial) correlations among our variables of interest with coefficient cutoffs of |r| = 0.10 indicating small, |r| = 0.30 medium, and |r| = 0.50 large correlations^52^. We compared correlation coefficients between male and female participants based on Fisher-z transformations^53^. Both MANOVA and correlation analyses were conducted with bias-corrected and accelerated bootstrapping (Bca; 5000 repetitions). In addition, we used Hayes’ PROCESS macro^54^ to conduct mediation (model 4) and moderated mediation (model 15) analyses. Z-standardized values were used. For (moderated) mediation analyses too, we applied bootstrapping with 5000 repetitions as well as heteroscedasticity-consistent standard errors (HC3: Davidson–MacKinnon) and considered respective confidence intervals (CI) which indicated significant results when not including zero-effects. Before these analyses, data were scanned for potential outliers by investigating data distribution to detect whether unplausible data values had been entered. This was, however, not the case. Beyond this, particularly high or low data values were not excluded or adapted as we aimed not to change the heterogeneity of the data, which was expected to be mostly non-normally distributed regarding the assessed construct.

## Results

### Descriptives

Table 1 displays the distribution of our variables of interest in the total sample and separately for male and female participants. MANOVA (controlled for age) indicated small but significant differences regarding higher ACE burden in females and more frequent use of suppression in males.

**Table 1:** Descriptive distribution of the variables of interest.

	Total sample (N = 287)				Males ( n = 130)				Females (n = 154)				*F(1,275)*	*p*	η_p_^2^
	M	SD	Range	Median	M	SD	Range	Median	M	SD	Range	Median			
MACE total score	19.33	16.58	0.00–78.25	15.25	17.07	13.64	0.00–68.33	15.08	21.39	18.61	0.00–78.25	17.45	5.54	0.019	0.02
ASR 18/59 aggressive behavior	6.10	5.49	0.00–25.00	5.00	6.07	5.28	0.00–25.00	5.00	6.19	5.70	0.00–23.00	5.00	0.07	0.792	0.00
ADHS-SB inattention	9.25	7.28	0.00–26.00	7.00	8.40	6.88	0.00–24.00	7.00	9.97	7.57	0.00–26.00	7.50	2.84	0.093	0.01
ADHS-SB hyperactivity/impulsivity	7.93	6.89	0.00–27.00	6.00	7.71	6.79	0.00–27.00	6.00	8.18	7.03	0.00–27.00	6.00	0.12	0.727	0.00
ERQ reappraisal	4.19	1.30	1.00–7.00	4.17	4.13	1.33	1.00–7.00	4.17	4.25	1.27	1.00–7.00	4.33	0.91	0.342	0.00
ERQ suppression	3.65	1.43	1.00–7.00	3.75	3.90	1.37	1.00–7.00	4.00	3.43	1.43	1.00–7.00	3.38	6.11	0.014	0.02

MANOVA was conducted with age as covariate. Due to missing values, subsample sizes were reduced to n = 128 males and n = 150 females in MANOVA.

*M* mean, *SD* standard deviation.

Table 2 represents correlational associations under statistical control of age and gender. Increasing ACE burden was moderately associated with higher aggression and ADHD severity on both dimensions, but not with emotion regulation. High positive correlations were found between both ADHD dimensions and aggression. Reappraisal showed moderate and negative associations with both ADHD dimensions and aggression. No significant correlations occurred with suppression.

**Table 2:** Partial correlations between the variables of interest with Bca 95% confidence intervals for the total sample.

	(1)	(2)	(3)	(4)	(5)	(6)
MACE total score (1)	–	0.383*** [0.281, 0.481]	0.293*** [0.176, 0.408]	0.320*** [0.2046 0.430]	− 0.069 [− 0.190, 0.048]	0.101 [− 0.021, 0.229]
ASR 18/59 aggressive behavior (2)		–	0.642*** [0.571, 0.706]	0.717*** [0.645, 0.781]	− 0.402*** [− 0.492, − 0.305]	0.021 [− 0.097, 0.139]
ADHS-SB inattention (3)			–	0.770*** [0.722, 0.813]	− 0.394*** [− 0.492, − 0.294]	0.068 [− 0.050, 0.192]
ADHS-SB hyperactivity/impulsivity (4)				–	− 0.321*** [− 0.425, − 0.214]	0.012 [− 0.107, 0.135]
ERQ reappraisal (5)					–	− 0.038 [− 0.170, 0.094]
ERQ suppression (6)						–

***p < 0.001. Analyses based on z-standardized values with age and gender as covariates. Due to missing values, sample size was reduced to N = 281.

Table 3 shows correlations among the abovementioned variables separately for male and female participants under the control of age. For females, the same associations appeared as for the total sample. For males, however, ACE burden was not related to the ADHD inattentional dimension, which was also underlined by the significant difference of the correlation coefficient compared to females.

**Table 3:** Partial correlations between the variables of interest with Bca 95% confidence intervals for males and females.

		(1)	(2)	(3)	(4)	(5)	(6)
MACE total score (1)	Male	–	0.296*** [0.174, 0.417]	0.113 [− 0.027, 0.251]	0.226* [0.090, 0.362]	0.029 [− 0.259, 0.301]	0.056 [− 0.121, 0.248]
	Female	–	0.421*** [0.313, 0.520]	0.380*** [0.258, 0.497]	0.362*** [0.235, 0.479]	0.138 [− 0.264, − 0.014]	0.139 [− 0.133, 0.363]
	Z	–	− 1.182	− 2.355*	− 1.226	− 0.903	− 0.689
ASR 18/59 aggressive behavior (2)	Male		–	0.581*** [0.386, 0.726]	0.683*** [0.595, 0.758]	− 0.365*** [− 0.549, − 0.159]	− 0.004 [− 0.226, 0.244]
	Female		–	0.675*** [0.541, 0.786	0.733*** [0.637, 0.814]	− 0.438*** [− 0.559, − 0.304]	0.029 [− 0.183, 0.234]
	Z		–	− 1.281	− 0.826	0.716	− 0.271
ADHS-SB inattention (3)	Male			–	0.732*** [0.607, 0.825]	− 0.380*** [− 0.519, − 0.234]	0.069 [− 0.192, 0.320]
	Female			–	0.796*** [0.715, 0.865]	− 0.402*** [− 0.565, − 0.210]	0.053 [− 0.200, 0.295]
	Z			–	− 1.270	0.213	0.132
ADHS-SB hyperactivity/impulsivity (4)	Male				–	− 345*** [− 0.544, − 0.122]	0.009 [− 0.242, 0.266]
	Female				–	− 0.311*** [− 0.438, − 0.173]	0.010 [− 0.235, 0.255]
	Z				–	− 0.313	− 0.008
ERQ reappraisal (5)	Male					–	− 0.038 [− 0.114, 0.184]
	Female					–	− 0.048 [− 0.179, 0.085]
	Z					–	0.082
ERQ suppression (6)	Male						–
	Female						–
	Z						–

*p < 0.05, **p < 0.01, ***p < 0.001. Analyses based on z-standardized values with age as a covariate. Due to missing values, sample size was reduced to n = 128 for males and n = 150 for females.

### Mediation analyses

**Fig. 1:**
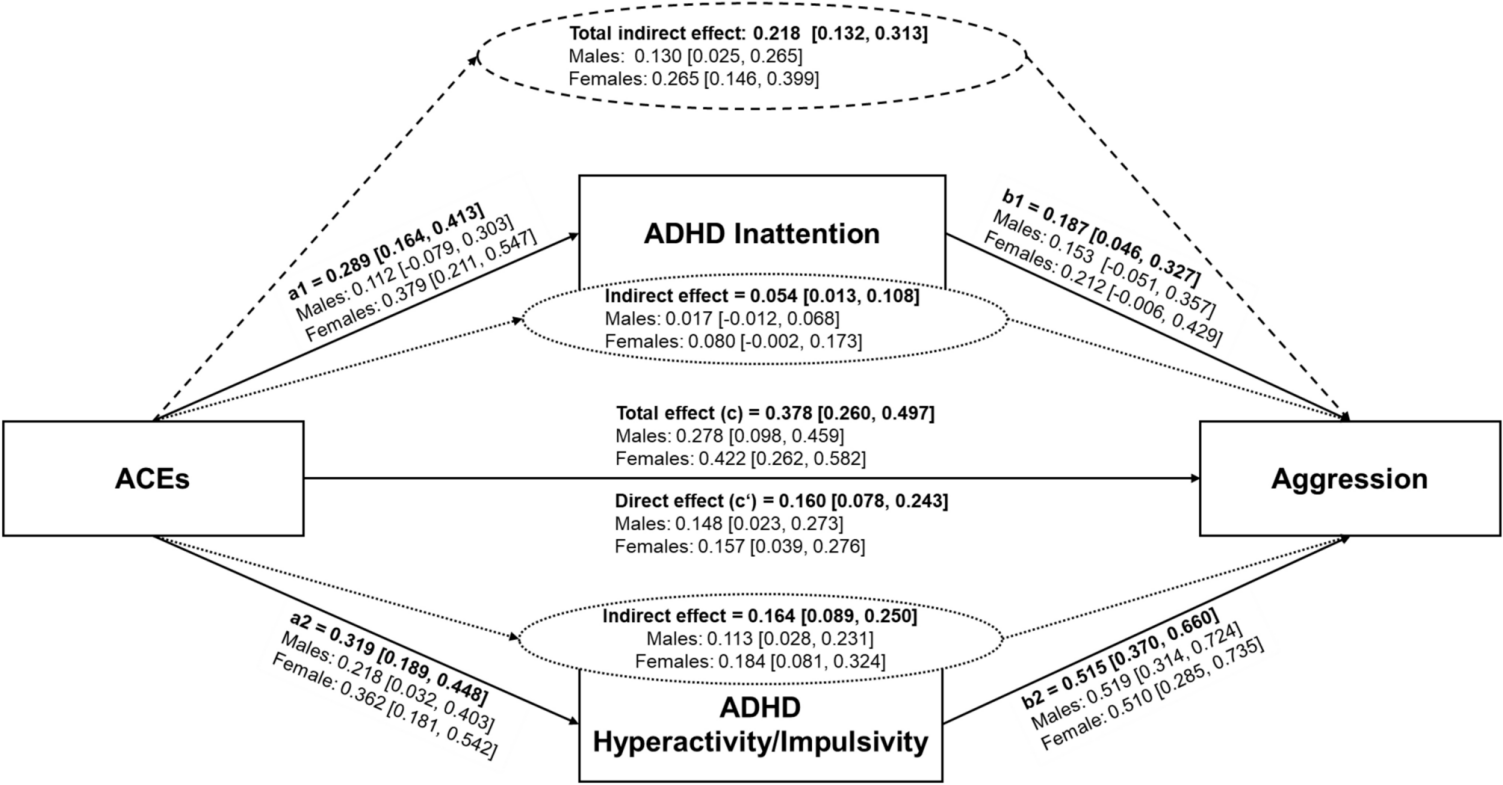
Mediation of the effect from ACEs on aggression over ADHD dimensions. For the total sample, analyses were controlled for age and gender. In the separated male and female models, age was included as a covariate. 95% confidence intervals in brackets.

Figure 1 displays the mediation model. When analyzing the total sample, age and gender were considered as covariates. This first model could explain 55.8% of the variance in the aggressive behavior variable (p < 0.001). The MACE score significantly predicted the ADHD inattention and hyperactivity/impulsivity dimensions as well as aggressive behavior. Moreover, both ADHD dimensions were significantly related to aggression. The direct effect of the MACE score on aggression was smaller than the total effect and a significant indirect effect indicated a partial mediation over both ADHD dimensions. Yet, the effect over the ADHD hyperactivity/impulsivity dimension was significantly larger than the effect over the ADHD inattention dimension, Δ =  − 0.110, SE = 0.048, 95% CI [− 0.208, − 0.021]. When males and females were separately considered (each under the control of age), models explained 52.1% of the variance in the aggressive behavior variable for males and 58.3% for females (p < 0.001). In both groups, there was a partial mediation over ADHD symptomatology as well, which was, however, mainly based on the hyperactivity/impulsivity dimension. Although the MACE score predicted inattention in females, no other paths involving ADHD inattention became significant. The indirect effect over ADHD inattention was significantly smaller than the effect over the ADHD hyperactivity/impulsivity dimension in males, Δ =  − 0.096, SE = 0.050, 95% CI [− 0.218, − 0.020], but not in females, Δ =  − 0.104, SE = 0.085, 95% CI [− 0.282, 0.046].

### Moderated mediation analyses

Eventually, we tested moderated mediation models for the total sample and gender-specific subsamples. We distinctively included reappraisal and suppression as multicategorical moderator variables. Based on the ERQ’s 7-point answer scheme, we considered mean values up to 3 as low, between 3 and 5 as moderate, and greater than 5 as high. The moderate category served as reference group. For the total sample (controlled for age and gender), reappraisal moderated the indirect effect over hyperactivity/impulsivity; the difference in conditional indirect effects between the moderate and the high reappraisal group was significant (Δ =  − 0.156, SE = 0.063, 95% CI [− 0.295, − 0.045]). The conditional indirect effect was highest for the moderate reappraisal group (B = 0.195, SE = 0.048, 95% CI [0.110, 0.298]), followed by the low reappraisal group (B = 0.146, SE = 0.066, 95% CI [0.025, 0.285]) and the high reappraisal group (B = 0.039, SE = 0.051, 95% CI [− 0.058, 0.140]), the latter not reaching statistical significance. There was no significant interaction on the direct path from ACEs to aggression and no moderation on the indirect effect over inattention. Suppression did not show any significant effects in moderation analyses.

When gender-based subsamples were distinctively analysed under the control of age, there was no indication of any moderation effect of reappraisal or suppression for males. However, in the female sample, similar dynamics appeared as in the non-gender specific model: Reappraisal moderated the indirect effect over hyperactivity/impulsivity; the difference in conditional indirect effects between the moderate and the high reappraisal group was significant (Δ =  − 0.163, SE = 0.100, 95% CI [− 0.398, − 0.002]). The conditional indirect effect was highest for the moderate reappraisal group (B = 0.228, SE = 0.076, 95% CI [0.096, 0.391]), followed by the low reappraisal group (B = 0.179, SE = 0.140, 95% CI [− 0.057, 0.499]) and the high reappraisal group (B = 0.065, SE = 0.082, 95% CI [− 0.103, 0.225]), the latter two not reaching statistical significance. There was no significant interaction on the direct path from ACEs to aggression and no moderation on the indirect effect over inattention. Suppression did not show any significant effects in moderation analyses for females.

## Discussion

Prior research has highlighted the bi-directional effects between ACEs and ADHD, the common co-appearance of maladaptive emotion regulation strategies and ADHD, and the role of each of these constructs for the risk of aggressive behavior^20,22,30^. However, since empirically based knowledge about the dynamics among ACEs, ADHD, aggression, and emotion regulation strategies was lacking, we conducted the current study to examine mediating and moderating effects in a mixed sample of adults.

In line with previous findings and our assumptions, ACEs were positively associated with adult aggression in our sample irrespective of age and gender^3,4,21,55^. Furthermore, ACEs were significantly related to more severe ADHD symptomatology. In particular, ACEs predicted higher levels of hyperactivity/impulsivity irrespective of gender, contributing to previous research that had related ACEs to the development of neurobiological, cognitive, and emotional deficits that may foster rather impulsive and unpremeditated behavior^27^.

With regard to gender differences, our findings contribute to prior research^2,3^ as females reported higher rates of ACEs than males. However, aggression and ADHD scores appeared comparable. Moreover, we could replicate studies that had found significantly higher rates of suppression as emotional regulation strategy in males than in females and a tendency of females to use cognitive reappraisal more often than males, which, however, did not reach statistical significance^39,49^. Additionally, ACEs were only associated with ADHD inattention for females but not for males. Haahr-Pedersen et al.^56^ found more versatile ACE patterns among females compared to males, suggesting that this difference in complexity could contribute to different long-term outcomes between genders. Although we did not examine ACE patterns in the present study, the significantly higher ACE severity of females compared to males may indicate similar assumptions.

The result that especially hyperactivity/impulsivity was associated with aggressive behavior and partially mediated its relationship with ACEs corresponds with earlier findings showing that individuals with mainly hyperactive/impulsive symptoms had a greater risk of aggression than those with mainly attention deficits^20,29^. The impact of high physical arousal for aggressive behavior has also been shown in studies regarding trauma-related disorders^57^. Among juvenile male offenders, the combination of hyperarousal and emotional numbing was the best predictor of serious aggressive and violent behavior^58^. Both aspects might also be prevalent in individuals affected by ADHD^59,60^. However, the role of inattention is less clear. Although there was a subtle indirect effect over inattention in the total sample, it was not remained in gender-specific analyses. The lower sample sizes in gender-separated subsamples might in part explain the statistical insignificance. However, the effect itself was small, thus calling into question the actual clinical significance of this result.

Cognitive reappraisal emerged in our study as the relevant emotion regulation strategy to reduce the indirect effect of ADHD hyperactivity/impulsivity on aggression, especially in females. This result is in line with research highlighting the importance of cognitive reappraisal to overcome emotional dysregulation in individuals affected by ADHD^61^. Cognitive reappraisal seems to improve their capacity to deal with aversive events and to reduce the therewith associated negative mood^62^, which might prevent aggression. However, ACEs were not associated with any of the two emotion regulation strategies examined in this study. This finding was surprising, considering the variety of research that has examined the associations between ACEs and emotional (dys-)regulation^63^. However, there is a wide heterogeneity when it comes to the assessment of ACEs and emotion regulation. Thus, different result may emerge depending on the definition of the constructs (e.g., emotion (dys-)regulation as a specific domain of psychopathology or coping strategy) and the instruments used.

As the present findings indicate that ADHD, particularly the hyperactive/impulsive dimension, seems to promote aggression, adequate treatment for ADHD may also reduce the risk of aggressive behavior. According to international recommendations, medication should be considered as first treatment option for adults with ADHD^64^. However, as there might be a delayed treatment effect on the hyperactive/impulsive dimension, risk of early treatment termination must not be neglected, thus underlining the need for psychosocial and psychological care as well^65^. These non-medical approaches could include coaching of adaptive emotion regulation strategies^34^. Although our correlational findings do not allow any causal interpretation, negative associations between cognitive reappraisal on the one hand and aggression and ADHD on the other hand indicate that the promotion of adaptive coping techniques may have favourable effects on aggression and ADHD severity. Moreover, Roberton et al.^36^ underlined that the improvement of emotion regulation skills can reduce aggressive behavior. However, further research is warranted, especially regarding the implementation and effects of adaptive emotion regulation strategies in males and females but also respective differences with regard to individuals with and without ADHD. Compared to females, male participants of the present study rather used suppression as a maladaptive emotion regulation technique. In contrast, Bodalski et al.^34^ stressed that females showed higher rates of maladaptive emotional coping related to emotional dysregulation in ADHD, but they also underlined the lack of clarity regarding differences in emotional coping between adults with and without ADHD in general. Rogier et al.^42^ found no independent effects of cognitive reappraisal or suppression on aggression for males, but concluded that the interaction of both strategies should be further examined.

Finally, as ADHD symptomatology is also related to ACEs, ACEs need to be taken into consideration in the treatment of ADHD as well. Since ACEs remained a robust predictor of aggressive behavior independent of ADHD and emotion regulation strategies, respective prevention and treatment approaches may also be fruitful to reduce the basic risk of aggressive behavior independent of ADHD symptomatology or emotional coping techniques.

The present study is not without limitations that need to be considered for interpretation and implications. First, although heterogeneous regarding age, gender, and assessment context, the present convenience sample only included individuals examined by one institute in Germany, thus impeding generalization of our results. Moreover, post-hoc power analyses indicated sufficient power (> 0.80) for analyses including the total sample, but not for (most) gender-specific analyses, which may explain the lack of significant findings in these subsamples and underlines the need to investigate larger samples in future research.

Moreover, data were based on self-reports and, at least for ACEs, on retrospective information, which bears the risk of bias. As the present study focused on self-reported current, dimensional adult ADHD symptomatology, there was no data on clinician-administered diagnosis for either childhood or current ADHD regarding ICD or DSM. Once again, it has to be underlined that the present study relied on cross-sectional data that cannot ensure that ADHD is a ‘true’, interposed mediator in terms of longitudinal development. Although the mediation models were based on obvious appearing theoretical assumptions about the developmental courses of ACEs, adult ADHD dimensions, and aggression, causal interpretations must not be drawn from this study but remain subject to future longitudinal research. Moreover, whereas theoretically expected, rather strong correlations between the ADHD dimensions and the aggression outcome raise the question of multicollinearity, which can lead to biased results. Although common indicators of multicollinearity were lying within acceptable limits (e.g., r < 0.80, VIF < 5, tolerance > 0.01^66^), clear interpretation of distinct effects remains complicated. Yet, by implementing parallel mediation instead of two separate mediation models for each ADHD dimension, we aimed to account for shared variance between the mediators, even if this may not have fully counteracted potential multicollinearity^54^. Apart from statistical considerations, the interplay among these constructs is not trivial at all, especially when considering potential genetic, neurological, or inter-generational influences that we could not include in the present study^6^. Furthermore, we did not control for potential medication effects or comorbid psychiatric conditions that might have influenced self-reports. In particular, comorbid symptoms of trauma-associated mental disorders such as (complex) post-traumatic stress disorder (PTSD) would need to be included in future studies that examine the link between ACEs, ADHD, and aggression^6^. We also considered only two strategies of emotion regulation, although further emotion but also stress coping techniques could be associated with our constructs of interest^67^.

In conclusion, our data support previous findings on the common co-occurrence of ACEs, ADHD, aggression, and emotion (dys-)regulation^20,36,55^, although the abovementioned limitations point to the need to further examine their associations, at best in large-scale longitudinal studies. In order to reduce their unfavourable dynamics and promote individual well-being of those affected by ACEs and ADHD, but also to decrease their risk of aggressive behavior, respective prevention and treatment should include both pharmacological and psychological approaches that not only consider the core symptoms of ADHD, but also emotion regulation techniques and the occurrence and potential consequences of ACEs.

## Data Availability

The data presented in this study are available on request from the first author (S.B.).
